# Modeling of auditory neuropathy spectrum disorders associated with the *TEME43* variant reveals impaired gap junction function of iPSC-derived glia-like support cells

**DOI:** 10.3389/fnmol.2024.1457874

**Published:** 2025-01-06

**Authors:** Xiaoming Kang, Lu Ma, Jie Wen, Wei Gong, Xianlin Liu, Yihan Hu, Zhili Feng, Qiancheng Jing, Yuexiang Cai, Sijun Li, Xinzhang Cai, Kai Yuan, Yong Feng

**Affiliations:** ^1^Department of Otorhinolaryngology, The Affiliated Changsha Central Hospital, Hengyang Medical School, University of South China, Changsha, China; ^2^Institute of Otorhinolaryngology, Head and Neck Surgery, University of South China, Changsha, China; ^3^MOE Key Lab of Rare Pediatric Diseases & Institute for Future Sciences, University of South China, Changsha, China; ^4^Institute of Cytology and Genetics, Hengyang Medical School, University of South China, Hengyang, China; ^5^Department of Otorhinolaryngology, Xiangya Hospital Central South University, Changsha, China; ^6^Hunan Key Laboratory of Molecular Precision Medicine, Department of Oncology, Xiangya Hospital & Center for Medical Genetics, School of Life Sciences, Central South University, Changsha, China

**Keywords:** auditory neuropathy spectrum disorder, TMEM43, glia-like support cells, gap junction, induced pluripotent stem cells, RNA-Seq

## Abstract

Auditory neuropathy spectrum disorder (ANSD) is an auditory dysfunction disorder characterized by impaired speech comprehension. Its etiology is complex and can be broadly categorized into genetic and non-genetic factors. *TMEM43* mutation is identified as a causative factor in ANSD. While some studies have been conducted using animal models, its pathogenic mechanisms in humans remain unclear. TMEM43 is predominantly expressed in cochlear glia-like support cells (GLSs) and plays a vital role in gap junction intercellular communication. In this work, we utilized induced pluripotent stem cells from an ANSD patient carrying the *TMEM43* gene mutation c.1114C>T (p.Arg372Ter) and directed their differentiation toward GLSs to investigate the effect of *TMEM43* mutation on the function of gap junctions in cochlear GLSs *in vitro*. Reduced expression of genes associated with GLSs characteristics and reduced gap junction intercellular communication in *TMEM43* mutant cell lines were observed compared to controls. Transcriptome analysis revealed that differentially expressed genes were significantly enriched in pathways related to cell proliferation, differentiation, extracellular space and adhesion. Furthermore, significant alterations were noted in the PI3K-Akt signaling pathway and the calcium signaling pathway, which could potentially influence gap junction function and contribute to hearing loss. In summary, our study based on patient-derived iPSCs sheds new light on the molecular mechanisms by which *TMEM43* mutations may lead to ANSD. These mutations could result in developmental defects in GLSs and a diminished capacity for gap junction function, which may be implicated in the auditory deficits observed in ANSD patients. Our study explored the pathological effects of the *TMEM43* mutation and its causal relationship with ANSD using a patient-derived iPSC-based GLSs model, providing a foundation for future mechanistic studies and potential drug screening efforts.

## 1 Introduction

Auditory neuropathy spectrum disorder (ANSD) is a dysfunctional disorder of the auditory nerve with the normal function of the outer hair cells and malfunction of the inner hair cells, ribbon synapses, spiral ganglion neurons, and/or the auditory nerve itself (Huang et al., 2022; Rance and Starr, 2015). The clinical manifestation of ANSD is characterized by mild hearing loss and, inconsistently, severely impaired speech comprehension. The audiogram exhibits an absence of auditory brainstem response (ABR) but normal otoacoustic emissions (DPOAE) and electrocochleography (ECoG) evoked summed potentials (SP; Kaga, 2016). ANSD is suggested to have different inheritance patterns, including syndromic which is associated with other disorders or non-syndromic which hearing loss is the only symptom (Saidia et al., 2023). Our previous study confirmed that the TMEM43-p.(Arg372Ter) mutation causes ANSD (Jang et al., 2021).

Transmembrane proteins (TMEM) are a family of transmembrane proteins that are widely involved in the structural composition of cell membranes and have a variety of functions, with important roles in the cellular, external environment and intercellular signaling, not only metabolic and immune roles but also important roles in tumorigenesis and development (Du and Lovly, 2018). The *TMEM43* gene is located at 3p25.1 and contains 12 exons with a total cDNA length of 3,230 bp. The encoded protein consists of 400 amino acids, has a molecular weight of 45 kDa, and its structure includes a large hydrophilic cytoplasmic structural domain, four transmembrane structural domains, several phosphorylation sites, a trans-activating structural domain, a SUMO linkage site, and O-glycosylation sites (Schirmer et al., 2003). In addition to ANSD, different mutations in *TMEM43* have been shown to cause arrhythmogenic right ventricular cardiomyopathy and Emory-Dreyfus muscular dystrophy (Liang et al., 2011; Hodgkinson et al., 2013).

The TMEM43 is predominantly expressed in glia-like supporting cells (GLSs) in the cochlea. The Organ of Corti of the inner ear consists mainly of inner hair cells, outer hair cells, and supporting cells. Hair cells play a key role in mechanoreception and synaptic transmission by converting acoustic energy into electrochemical signals transmitted to the brainstem, and GLSs are located in the vicinity of the hair cells and also play an important role in the development and maintenance of the auditory system (Wang et al., 2015; Jang et al., 2022). Although it has been demonstrated in animal models that TMEM43-p.(Arg372Ter) mutation disrupts the function of gap junction proteins in cochlear GLSs, affects K^+^ cycling, and exhibits progressive hearing impairment, its pathogenic mechanism remains poorly characterized in humans (Jang et al., 2021). Induced Pluripotent Stem Cell (iPSC) technology is a leap forward in experimental modeling of human diseases. The use of iPSCs to differentiate into disease-related target cells and to mimic the molecular and cellular phenotypes of specific genotypes has far-reaching significance for understanding the pathogenesis of diseases and precision therapy (Kumar et al., 2018; Shevade et al., 2023). Methods of induced generation of cells with cochlear support cell characteristics using human iPSCs have been reported in the literature (Fukunaga et al., 2021). To determine whether *TMEM43* mutations lead to alterations in the specific function of GLSs, we utilized iPSCs of patients with *TMEM43* mutation to establish a model for the induction of GLSs.

In this study, we generated a patient-specific *TMEM43* mutant iPSCs and differentiated it into GLSs to study how *TMEM43* mutations affect the development and function of GLSs. The many differences in molecular characterization and gap junction function between patient-derived GLSs and normal controls suggest that *TMEM43* mutations affect the development and function of cochlear support cells. In addition, to better understand the pathogenic mechanism of ANSD caused by TMEM43-p.(Arg372Ter) mutation, we performed transcriptome sequencing of differentiated GLSs to search for the potential molecular mechanisms of the differentially expressed genes (DEGs).

## 2 Materials and methods

### 2.1 Generation and maintenance of patient-derived iPSCs from TMEM43 mutant ANSD

Generation of iPSCs using peripheral blood mononuclear cells (PBMCs) from the ANSD patient and his healthy daughter. The PBMCs were cultured in 1640 (Gibco, United States) supplemented with 25% fetal bovine serum (FBS; Sigma, Germany), 2 mM L-glutamine (Invitrogen, United States) at 37°C in 5% CO_2_. The PBMCs (2 × 10^6^cells) were electroporated with 2 μg per vector of the following five episomal vectors pCXLE-hUL, pCXLE-hSK, pCXLE-hOCT3/4-shp53-F, pCXWB-EBNA1, pCXLE-EGFP on Nucleofector™ 2b device (Lonza, Germany) using Kit VCA-1003 (Lonza, Germany). After electrotransformation cells were cultured in 12-well plates with WiCell + VitC medium (DMEM/F12; Gibco, United States) supplemented with 20% KO serum replacer (Gibco, United States), 1% GlutaMax (Gibco, United States), 1% non-essential amino acids (Gibco, United States), 10 ng/mL bFGF (R&D, United States), 0.1 mm *β*-mercaptoethanol (Sigma, Germany) and 50 μg/mL VitC (Sigma, Germany), and the solution was changed every 2 days. From day 2 to 12, 0.5 mM sodium butyrate (Sigma, Germany) was added to the medium. On day 8, cells were transferred to inactivated mouse embryonic fibroblast-coated 6-well plates (Corning, United States). On days 16–28, iPSC clones were visible under the microscope and different clones were selected for amplification. The iPSCs were cultured in mTeSR™1 medium (Stemcell, Canada) on Matrigel®Matrix (Corning, United States)-coated plates. The iPSCs were digested with Accutase (Gibco, USA) every 4–5 days and then routinely passaged at a 1:6 ratio. A 10 μM concentration of ROCK inhibitor (#Y27632, MCE, United States) was added for the first 24 h after each passaging (Li et al., 2022).

### 2.2 Sanger sequencing

The iPSCs DNA was extracted using the Tissue gDNA Miniprep kit (Biomiga, United States) and PCR amplification reactions were performed on the *TMEM43* c.1114C>T variant region (GGTTTCCTGTTTTCCGAGAC/GTCAGCTTGCCATTCATGAG) using 2 × Master Mix (Yeasen, China). PCR products were analyzed by Sanger sequencing (Tsingke, China).

### 2.3 Alkaline phosphatase staining

The iPSCs were washed twice with phosphate-buffered saline (PBS; Gibco, United States), added with an appropriate amount of 4% paraformaldehyde (PFA; Servicebio, China), and fixed for 20 min at room temperature away from light, then washed three times with PBS and incubated with alkaline phosphatase coloring reagent (Biyun Tian, China) at room temperature away from light for 1–2 h.

### 2.4 Immunofluorescence staining

At room temperature, the cells were wetted with PBS and fixed in 4% paraformaldehyde for 20 min, and then permeabilized with 1% Triton X-100 (Sigma, Germany) for 10 min. Subsequently, the cells were lubricated with PBS three times and closed with 5% bovine serum albumin (BSA; Sangon Biotech, China) for 1 h. Cells were then incubated overnight at 4°C using primary antibodies in a PBS solution containing 2% BSA. On the second day, the cells were washed three times with PBS and then incubated with secondary antibodies in PBS solution containing 2% BSA at room temperature for 1 h. The last, nuclei were restained with 4′,6-diamino-2-phenylindole (DAPI; Beyotime, China). A STELLARIS 8 (Leica) confocal microscope was used for imaging photographs. Supplementary Table S1 details the antibodies used in this study.

### 2.5 Quantitative reverse transcription-PCR

Total RNA from cells was extracted by ReliaPrep RNA Miniprep Systems (Promega, United States) and then reverse transcription into cDNA by using the PrimeScript™ RT reagent Kit (Takara, Japan). 2 × TOROGreen® qPCR Master Mix (TOROIVD, China) was used to perform qRT-PCR system on the reverse transcription products. We used GAPDH as an internal control gene and calculated the relative expression of the target genes using the 2^-△△Ct^ method. Repeat at least 3 times for each sample. Details of the primers used are shown in Supplementary Table S2.

### 2.6 Karyotyping

The two iPSCs were sent to Hunan Jiahui Genetic Hospital (Changsha, Hunan, China) for karyotyping. Each iPSC line was analyzed with at least 20 metaphases (400 band resolution).

### 2.7 Teratoma experiments

Accutase digestion was performed to collect iPSCs (1 × 10^7^ cells) and suspended in 100 μL of 0.5 × Matrigel (Corning, United States), which was then injected into the dorsolateral region of NOD/SCID mice (Cavens, Changzhou, China). 8–10 weeks after injection, the teratoma was dissected, fixed in 4% PFA, embedded in paraffin, and finally stained with hematoxylin–eosin.

### 2.8 Induction of GLSs from iPSCs

Differentiation of GLSs from human iPSCs using established protocols (Fukunaga et al., 2021). We cultured the iPSCs in mTeSR medium (Stemcell, Canada) on Matrigel-coated 6-well plates until passage 15. We used 15th-20th generation iPSC cells for induced differentiation. The iPSCs were isolated with Accutase, suspended in mTeSR™1 medium supplemented with Y-27632 (20 μM), and then cultured at 100 μL/well (9,000 cells) in 96-well low-cell-attachment U-bottom (Corning, United States) for 2 days. The cells were transferred to a new 96-well low-cell-attachment U-bottom medium and cultured for 7–11 days with growth factor-free chemically defined medium (gfCDM) supplemented with 2% Matrigel (Corning, United States) and 7ug/ml insulin (MCE, United States). The gfCDM contained Ham’s F12 GlutaMax (Gibco, United States), IMDM GlutaMax (Gibco, United States), 1% Chemically Defined Lipid Concentrate (Gibco, United States), 0.5% BSA (Sigma, Germany), 15ug/ml Transferrin (Sigma, Germany), 450 uM 1-Thioglycerol (Sigma, Germany). On days 7–11 of differentiation, we separated the vesicles from the aggregates with forceps and transferred them to inactivated trypsin-resistant inner ear cells (TRICs; 3 × 10^5^/cm^2^), which were cultured in 24-well plates in adherent culture with DFNB medium for 7 days. TRICs were derived from the cochlear cells of 10-week-old mice. The DFNB medium contained DMEM/F12 (Gibco, United States) supplemented with 1% N-2 (Gibco, United States) and 2% B-27 (Gibco, United States). The medium was then changed to DMEM glutaMAX (Gibco, United States) with 10% FBS to continue culturing for 7 days.

### 2.9 Transmission electron microscopy

Differentiated GLSs (2 × 10^6^) were collected with a cell scraper and fixed overnight at 4°C with 2.5% glutaraldehyde (Solarbio, China). Firstly, the samples were washed three times with Millonig’s phosphate buffer for 10 min each time. Incubate in 1% osmium tetroxide for 1 h, then wash 3 times for 10 min each with Millonig’s phosphate buffer. Secondly, the samples were graded dehydration with 50, 70 and 90% acetone for 10 min each at room temperature, followed by two 15-min sessions with 100% acetone. Thirdly, the samples were soaked in a 1:1 mix of acetone: resin for 12 h and 100% resin embedded overnight at 37°C. Fourthly, samples were oven-cured at 37°C overnight and then 60°C for 12 h. Leica UC-7 Ultrathin Slicer Sections 50-100 nm. Finally, the samples were double-stained with 3% uranyl acetate and lead nitrate and placed under a Hitachi HT-7700 electron microscope for photographs.

### 2.10 Scratch loading/dye transfer analysis

The Scratch loading/Dye transfer (SL/DT) detection methods were as previously described (Yum et al., 2010; Fukunaga et al., 2019). Differentiated GLSs were subjected to SL/DT experiments, while undifferentiated iPSCs and TRICs served as controls. The cells were rinsed with PBS and covered with a sufficient amount of 0.1% Lucifer yellow CH (Thermo, United States). A razor blade was used to randomly draw 3 parallel lines through the cells in the dish. Protect from light and incubate at room temperature for 10 min and then wash with PBS 3 times for 3 min each time. Then fixed with 4%PFA and imaged. We quantified the extent of dye transfer by measuring the vertical distance from the scratch light to the point where the fluorescence intensity drops to 1.5 × the background fluorescence intensity. Images were processed and analyzed using NIH ImageJ software and statistically analyzed using GraphPad Prism 9 software.

### 2.11 RNA sequencing and data analysis

Total RNA from 2 groups of GLSs on day 24 of differentiation was extracted for RNA-Seq, and each group was repeated 3 times. Total RNA was isolated using Animal Total RNA Extraction Kit (Magnetic Bead Method) #T102096. Paired-end libraries were synthesized by using the TruSeq® RNA Sample Preparation Kit (Illumina, United States) following TruSeq® RNA Sample Preparation Guide. Briefly, The poly-A containing mRNA molecules were purified using poly-T oligo-attached magnetic beads. Following purification, the mRNA is fragmented into small pieces using divalent cations under 94°C for 8 min. The cleaved RNA fragments are copied into first strand cDNA using reverse transcriptase and random primers. This is followed by second strand cDNA synthesis using DNA Polymerase I and RNase H. These cDNA fragments then go through an end repair process, the addition of a single ‘A’ base, and then ligation of the adapters. The products are then purified and enriched with PCR to create the final cDNA library. Purified libraries were quantified by Qubit® 2.0 Fluorometer (Life Technologies, United States) and validated by Agilent 2,100 bioanalyzer (Agilent Technologies, United States) to confirm the insert size and calculate the mole concentration. Cluster was generated by cBot with the library diluted to 10 pM and then were sequenced on the Illumina Noveseq 6,000 (Illumina, United States). The library construction and sequencing was performed at Shanghai Biotechnology Corporation. Sequencing raw reads were preprocessed by filtering out rRNA reads, sequencing adapters, short-fragment reads and other low-quality reads. We used HISAT2 (version: 2.0.4) to map the cleaned reads to the GRCh38 reference genome with two mismatches. After genome mapping, StringTie (version: 1.3.0) was run with a reference annotation to generate FPKM values for known gene models. Differentially expressed genes were identified using Stringtie. The *p*-value significance threshold in multiple tests was set by the false discovery rate (FDR). The fold-changes were also estimated according to the FPKM in each sample. Differentially expressed genes (DEGs) were screened by adjusting |log2FC|≧1, Q≦0.05. Gene ontology (GO) and Kyoto Encyclopedia of Genes and Genomes (KEGG) pathway enrichment analysis of all DEGs. Adjusted *p* < 0.05 indicates significant enrichment. ANSD patient and his healthy daughter are different in sex. To eliminate sex-based biases, we excluded excluding genes located on the sex chromosomes.

### 2.12 Statistical analyses

The iPSCs were differentiated at least three times independently and all experiments were repeated at least three times. The results are expressed as the mean ± standard. The statistical difference was determined by Scheffe’s multiple comparison test. Images were processed and analyzed using NIH ImageJ software and statistically analyzed using GraphPad Prism 9 software. ****p < 0.0001; ***p < 0.001; **p < 0.01; *p < 0.05; n s, no significant difference.

## 3 Results

### 3.1 Somatic induced iPSCs from patients with ANSD

**Figure 1:**
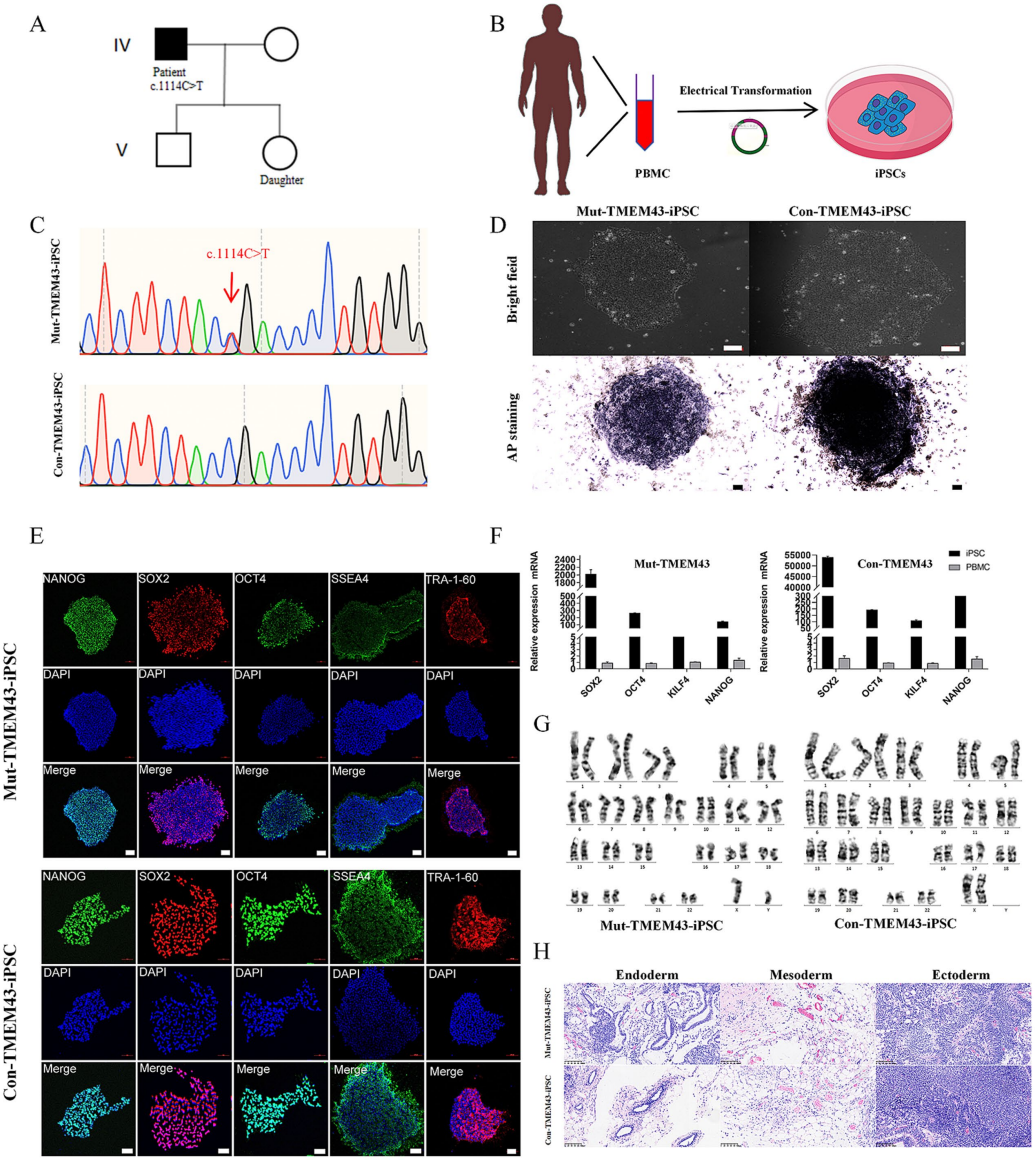
Induced generation of iPSCs from ANSD patient and healthy daughter as controls. **(A)** Partial lineage map and sequencing results of the patient. **(B)** Schematic illustration that PBMCs were isolated from the blood of ANSD patients and iPSCs were generated by electrotransferring stem plasmids. **(C)** Representative DNA sequencing results from Mut-TMEM43-iPSCs. **(D)** Morphology of Mut and Con-iPSCs (upper panel) and AP staining (lower panel), bar = 100 μm. **(E)** Immunofluorescence staining was performed for the pluripotency transcription factors NANOG, SOX2, OCT4 and the cell-surface markers SSEA4 and TRA-1-60, bar = 100 μm. **(F)** Expression of pluripotency markers in Mut-TMEM43-iPSCs and Con-TMEM43-iPSCs, as determined by qRT-PCR, *n* = 3. **(G)** Karyotype of Mut-TMEM43-iPSC and Con-TMEM43-iPSC. **(H)** Mut-TMEM43-iPSC and Con-TMEM43-iPSC teratoma histology, bar = 100 μm.

For this study, we selected a patient with ANSD should be (IV-1) and his normal daughter (V-2) from the family of ANSD obtained in our previous study (Jang et al., 2021) and obtained their blood samples (Figure 1A). Generation of iPSCs using PBMCs from the ANSD patient and his healthy daughter (Figure 1B). PBMCs (2 × 10^6^ cells) were electroporated with 2 μg per vector of the following five episomal vectors pCXLE-hUL, pCXLE-hSK, pCXLE-hOCT3/4-shp53-F, pCXWB-EBNA1, and pCXLE-EGFP (Okita et al., 2011). Post-induction iPSCs carrying *TMEM43* mutation were confirmed by Sanger sequencing (Figure 1C). A short tandem repeat (STR) analysis showed that both iPSC lines were identical to their peripheral blood mononuclear cells, with a 100% match (data not shown). Both Mut-TMEM43-iPSCs and Con-TMEM43-iPSCs have typical iPSC colony morphology under a light microscope, and positive alkaline phosphatase staining confirms the undifferentiated state (Figure 1D, bar = 100 μm). These two iPSC lines were verified to express five pluripotency markers (NANOG, SOX2, OCT4, SSEA4, and TRA-1-60) by immunofluorescence staining (Figure 1E, bar = 100 μm). In addition, quantitative reverse transcription PCR (qRT-PCR) further confirmed that the expression of SOX2, OCT4, KILF4, and NANOG was significantly elevated in iPSC lines compared to PBMCs (Figure 1F). Karyotype analysis showed that these two iPSC lines had normal karyotypes and chromosomal stability (Figure 1G). Furthermore, the teratoma-forming experiments confirm that two iPSC lines could differentiate into all three germ layers (Figure 1H). After these two iPSC lines were cultured through 15–20 passages, PCR showed that the exogenous plasmid inserted by electrotransfer had completely disappeared (Supplementary Figure S1).

### 3.2 TMEM43-Mut ANSD patient-derived iPSCs differentiate into GLSs with gap junctions

**Figure 2:**
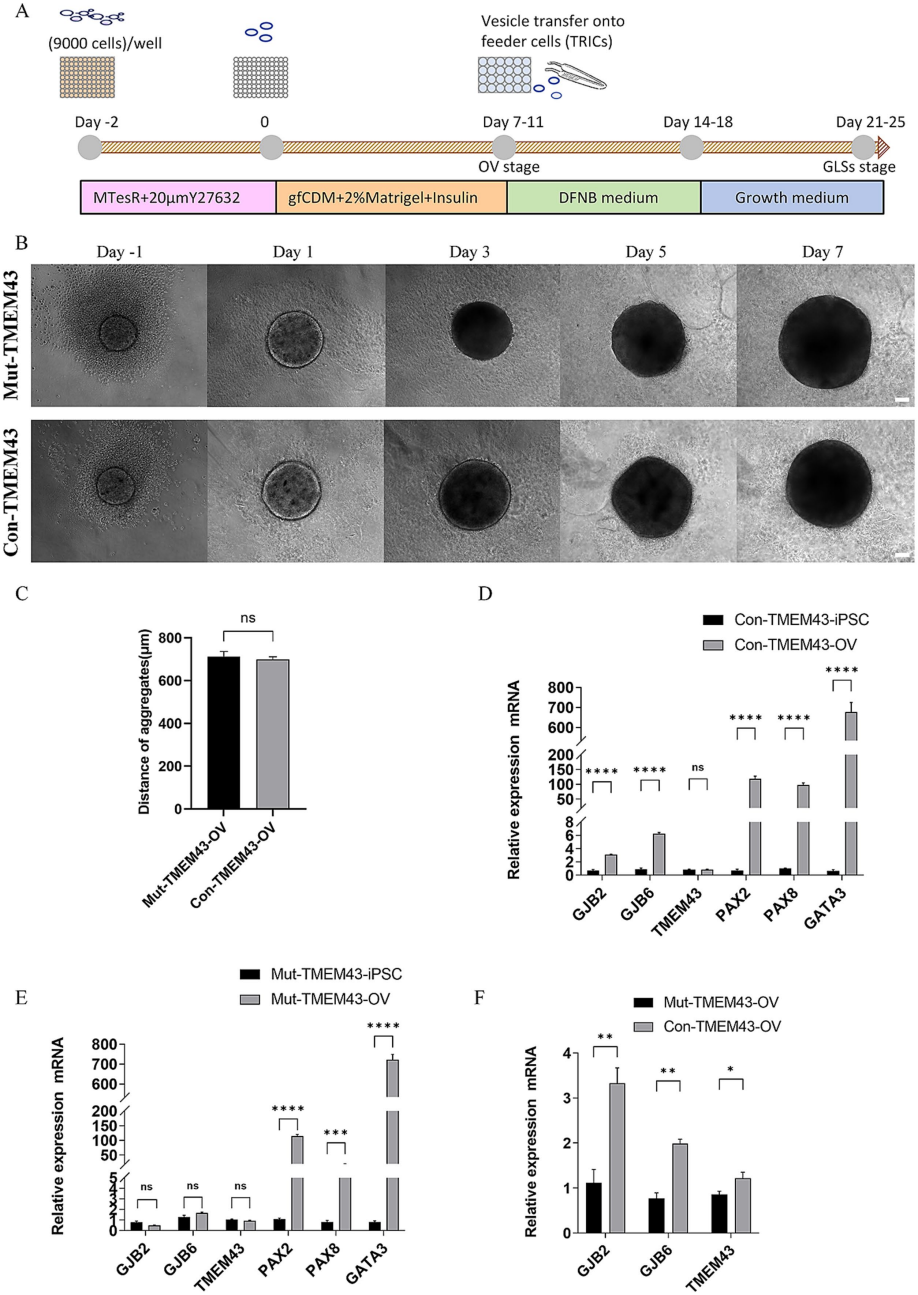
Differentiation of Mut-TMEM43-iPSC and Con-TMEM43-iPSC toward otic vesicle cells. **(A)** Procedure for the differentiation of GLSs from human iPSCs. **(B)** Comparison of cell morphology during differentiation of Mut-TMEM43-iPSC and Con-TMEM43-iPSC to otic vesicle cells. **(C)** The statistical plot of Mut-TMEM43-OV and Con-TMEM43-OV diameters at 7 days of differentiation, n = 10, (ns, no significance). **(D–F)** Quantitative RT-PCR analysis of GJB2, GJB6, TMEM43, PAX2, PAX8 and GATA3 expression in undifferentiated human iPSCs (Day 0) and iPSC-derived otic vesicles (Day 7), *n* = 3, (****p < 0.0001, ***p < 0.001, **p < 0.01, *p < 0.05, and ns, no significance).

To further investigate the effect of TMEM43 mutations on cochlear GLSs, we differentiated Mut-TMEM43-iPSCs and Con-TMEM43-iPSCs into GLSs with gap junction function (Mut-TMEM43-GJCs and Con-TMEM43-GJCs; Figure 2A).

#### 3.2.1 Characterization of OV stage during differentiation

The iPSCs were maintained in MTesR medium in 96-well low-cell-attachment U-bottom 3D suspension culture for 2 days and the appearance of aggregates was seen. These aggregates were transferred to a new 96-well low-cell-attachment U-bottom and induced to culture with gfCDM (containing 2% Matrigel and 7ug/ml Insulin) for 7–11 days. These aggregates have many vesicular structures which have been shown to highly express CX26 and CX30 (Fukunaga et al., 2021; Fukunaga et al., 2019). No difference in morphology and diameter size between Mut-TMEM43-OV (713.2 ± 72.8um) and Con-TMEM43-OV (699.4 ± 37.9um) during differentiation (Figures 2B,C). On day 7, qRT-PCR analysis showed that both aggregates expressed high mRNA transcript levels of otic progenitor cell markers, including *PAX2*, *PAX8*, and *GATA3* genes, compared to undifferentiated iPSCs. The *GJB2*, *GJB6* and *TMEM43* genes are mainly expressed in cochlear support cells. No significant changes in *TMEM43* mRNA transcript levels were seen in both sets of aggregates on day 7 of differentiation. However *GJB2* and *GJB6* mRNA transcript levels were significantly higher in aggregates from normal controls compared to iPSCs, but not in the TMEM43 mutant group. Expression levels of *GJB2*, *GJB6* and *TMEM43* genes were statistically different between the two groups of aggregates (Figures 2D–F).

#### 3.2.2 Characterization of glia-like support cells stage during differentiation

**Figure 3:**
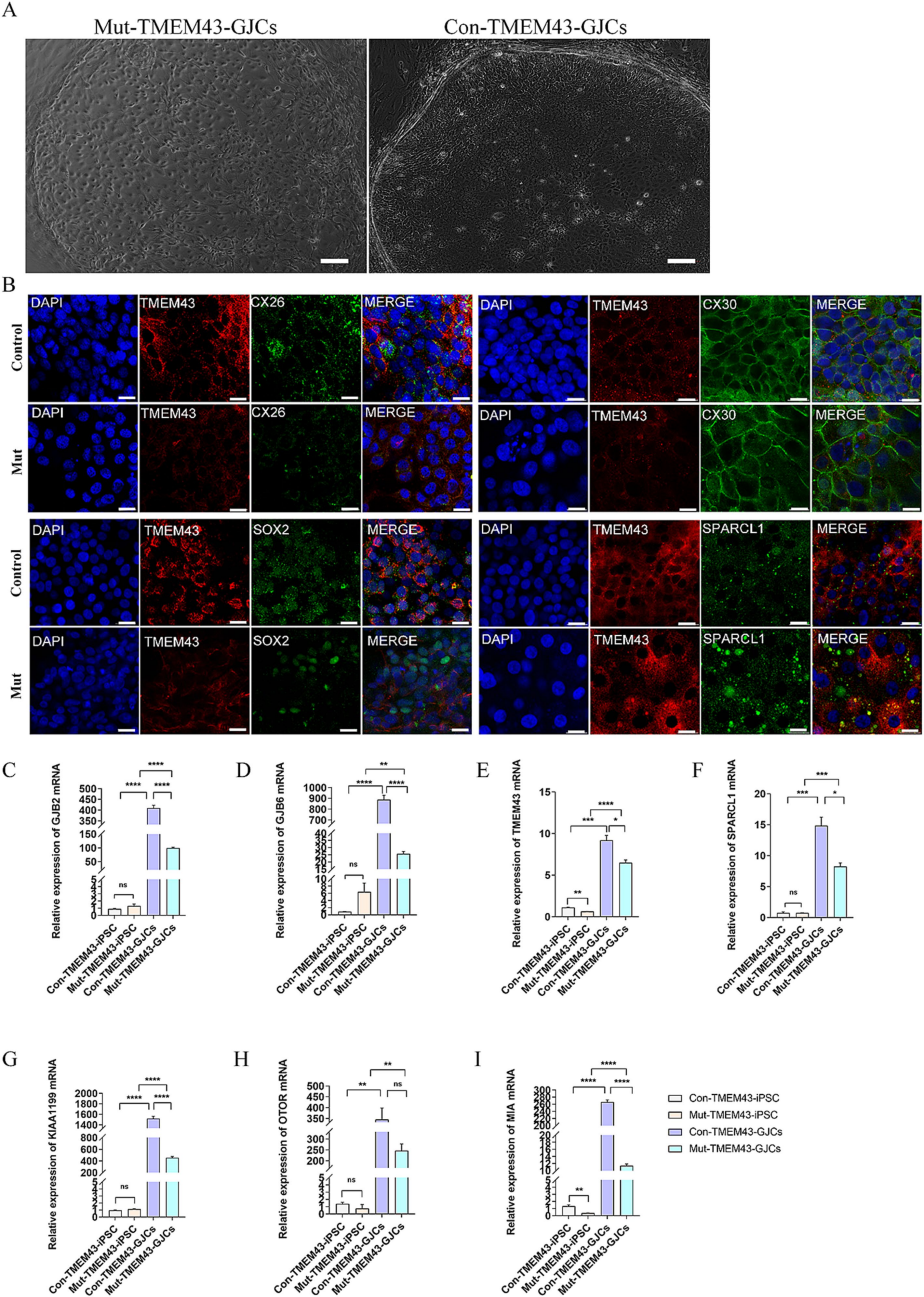
Differentiation of Mut-TMEM43-iPSC and Con-TMEM43-iPSC toward GLSs. **(A)** Comparison of cell morphology between Mut-TMEM43 and Con-TMEM43 on day 24 of differentiation to GLSs. **(B)** Immunofluorescence staining for GLSs markers TMEM43, CX26, CX30, SOX2, SPARCL1, bar = 20 μm. **(C–I)** Quantitative RT-PCR of undifferentiated human iPSCs and Mut-TMEM43-GJCs, Con-TMEM43-GJCs to analyze the expression of *GJB2*, *GJB6*, *TMEM43*, *SPARCL1*, *KIAA1199*, *OTOR* and *MIA*, *n* = 3, (****p < 0.0001, ***p < 0.001, **p < 0.01, *p < 0.05, and ns, no significance).

On day 7–11 of differentiation, we separated the vesicles from the aggregates with forceps and transferred them to inactivated TRICs, which were cultured in 24-well plates in adherent culture with DFNB medium for 7 days. The medium was then changed to a growth medium to continue the culture for 7 days. On day 24 of differentiation, vesicles were seen to form larger colonies on TRICs under light microscopy, and the cells in the control colonies appeared to be more compact (Figure 3A). To clarify whether these colonies resemble GLSs in the human cochlea, we performed immunofluorescence validation to confirm that these two groups of cells co-express the proteins GJB2, GJB6, TMEM43, SOX2, SPARCL1, PAN and CK18, which were found in mammalian cochlear supporting cells (Figure 3B; Supplementary Figure S2; Fukunaga et al., 2021). The qRT-PCR assay also showed that cells on day 24 of differentiation highly expressed the support cell marker genes *GJB2*, *GJB6*, *TMEM43*, *SPARCL1*, *KIAA1199*, *MIA*, and *OTOR* compared to iPSCs, which is a further indication of success in inducing GLSs. To study the function of TMEM43 gap junctions, we defined these GLSs as TMEM43 gap junction forming cells (TMEM43-GJCs). The mRNA transcript levels of *GJB2*, *GJB6*, *KIAA1199*, and *MIA* genes were significantly decreased in Mut-TMEM43-GJCs compared to Con-TMEM43-GJCs, suggesting that the TMEM43 mutation affects the expression and trans-activation of these genes. However, the mRNA transcript levels of *TMEM43*, *SPARCL1* and *OTOR* genes did not differ significantly between the two groups of differentiated cells (Figures 3C–I). Thus, TMEM43 mutations directly affect the proliferation and differentiation of iPSCs into GLSs.

#### 3.2.3 Decreased gap junction intercellular communication in Mut-TMEM43-GJCs

**Figure 4:**
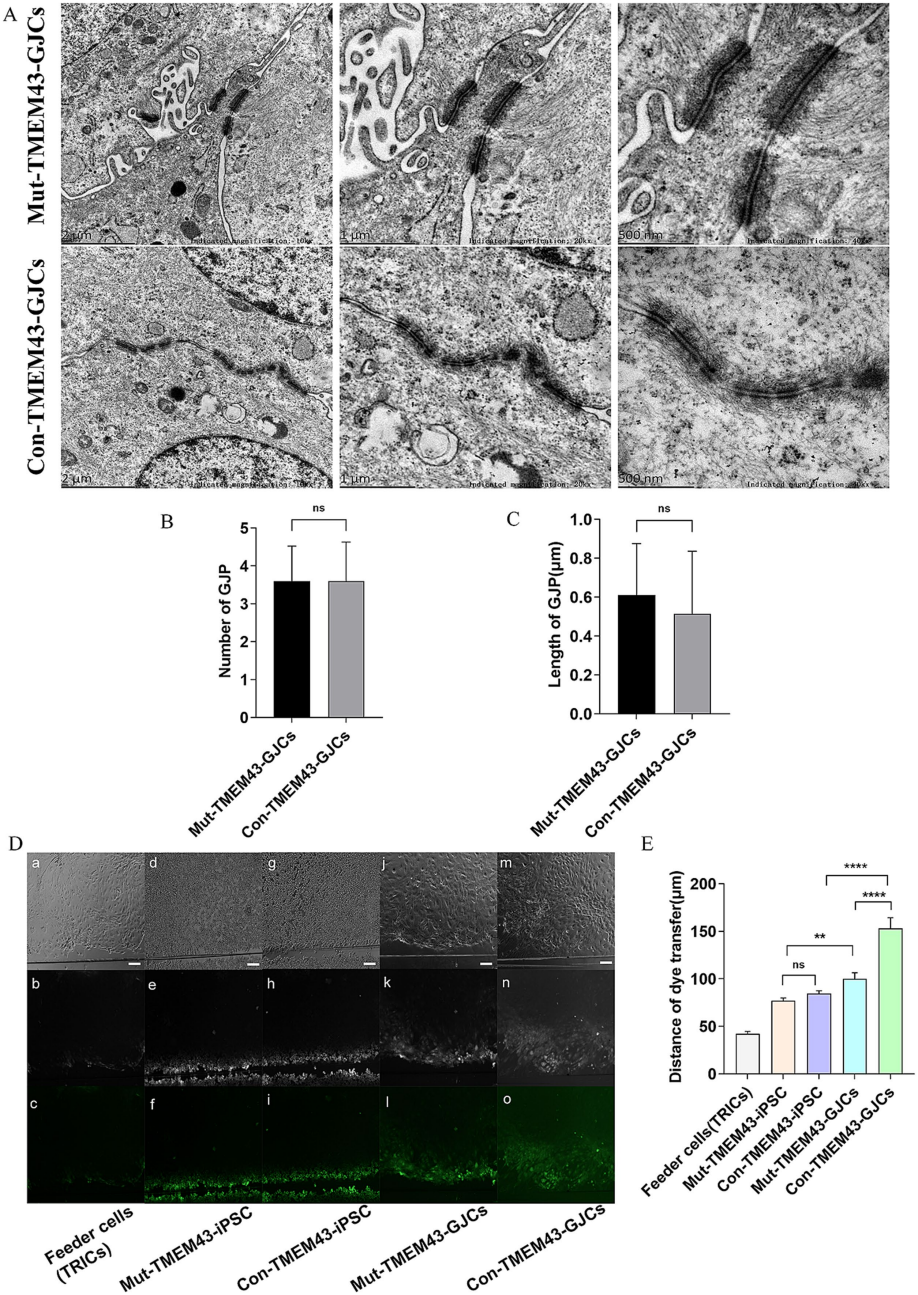
Mut-TMEM43-GJCs gap-junction function reduced. **(A)** Mut-TMEM43-GJCs and Con-TMEM43-GJCs have typical GJPs under transmission electron microscopy observation. Scale bars represent 2 μm, 1 μm and 500 nm, respectively. **(B)** Number of gap junctions per 5Kx transmission electron microscope field of view, *n* = 5, (ns, no significance). **(C)** Length of GJPs, *n* = 13, (ns, no significance). **(D)** Digital fluorescence images of cultured cells after scrape loading. (a–c) Feeder cells (TRICs). (d–f) Mut-TMEM43-iPSC. (g–i) Con-TMEM43-iPSC. (j–l) Mut-TMEM43-GJCs. (m–o) Mut-TMEM43-GJCs. (a, d, g, j, m) Phase contrast microscopy, bar = 100 μm. (b, e, h, k, n) Dye transfer using LY. (c, f, i, l, o) Pseudocolor images indicate the range of transfer from low to high signal intensity in the same region as shown in the above images. **(E)** Quantitative analysis of intercellular dye transfer after scrape loading. Columns represent the mean distance of dye transfer from the scrape line, *n* = 40, (****p < 0.0001, **p < 0.01, and ns, no significance).

We analyzed the ultrastructure of TMEM43-GJCs in cellular junction sites by transmission electron microscopy (TEM) on day 24 of differentiation. We found that iPSCs differentiation-derived cells have typical gap junctions plaques (GJPs; Figure 4A). The number and length of GJPs of Con-TMEM43-GJCs and Mut-TMEM43-GJCs were not statistically significant in the 5 K magnification field of view of TEM (Figures 4B,C). To investigate the function of the communication network we observed between cellular gap junctions, we performed SL/DT experiments on TRIC feeder cells, undifferentiated iPSCs, Mut-TMEM43-GJCs and Con-TMEM43-GJCs. TRIC feeder cells and undifferentiated iPSCs were used as controls. We quantified the extent of dye transfer by measuring the vertical distance from the scratch light to the point where the fluorescence intensity drops to 1.5 × the background fluorescence intensity. In cultures of these differentiated cells, we observed LY (Lucifer yellow) spreading beyond the injured parental cells, suggesting that these differentiated cells function as gap junctions. However, this dye diffusion was not observed significantly in TRIC feeder cells and undifferentiated iPSCs (Figure 4D). The quantitative distance of dye transfer in Mut-TMEM43-GJCs (99.9 ± 6.6um), Con-TMEM43-GJCs (153.3 ± 10.9um) was significantly longer than that in Mut-TMEM43-iPSCs (77.0 ± 3.0um), Con-TMEM43-iPSCs (84.5 ± 3.1um), TRIC feeder cell (42.3 ± 2.4um) (Figure 4E). In addition, the dye transfer distance of Mut-TMEM43-GJCs was significantly shorter than that of Con-TMEM43-GJCs, suggesting that the *TMEM43* mutation reduced the function of intercellular gap junctions.

#### 3.2.4 Transcriptome analysis of GLSs inducing differentiation of ANSD patient-specific iPSCs

**Figure 5:**
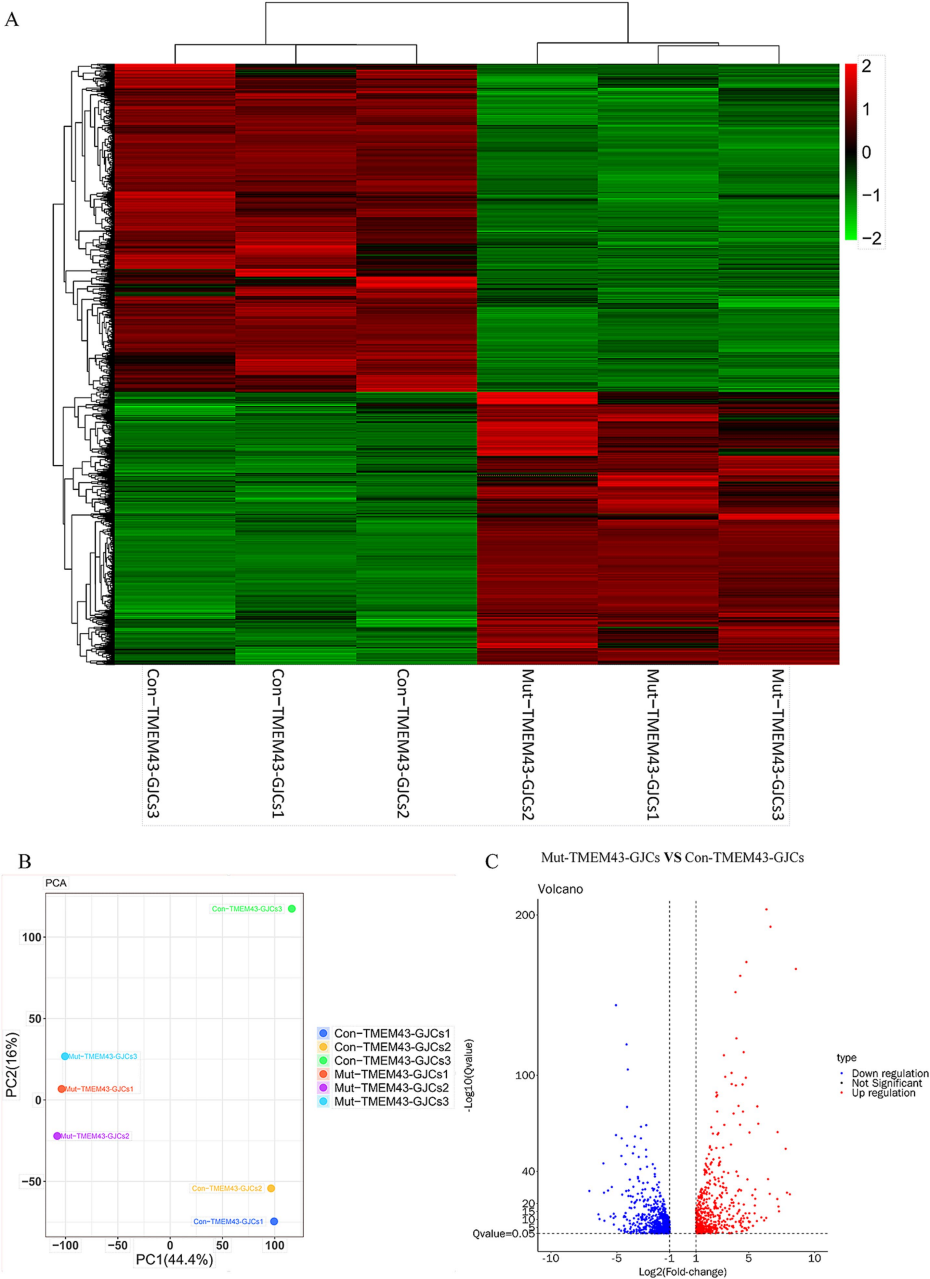
RNA sequencing (RNA-seq) analysis of Mut-TMEM43-GJCs and Con-TMEM43-GJCs. **(A)** Heatmap showing hierarchical clustering analysis of differentially expressed genes (DEGs) in Mut-TMEM43-GJCs and Con-TMEM43-GJCs. The expression values of the DEGs were normalized by a scaling function and compared between these two cell lines (each group contains 3 samples). Red and green colors indicate genes with high and low expression levels, respectively. **(B)** Principal component analysis (PCA) of two sets of RNA-seq data. **(C)** Comparison of volcano mapping of Mut-TMEM43-GJCs and Con-TMEM43-GJCs differential genes.

**Figure 6:**
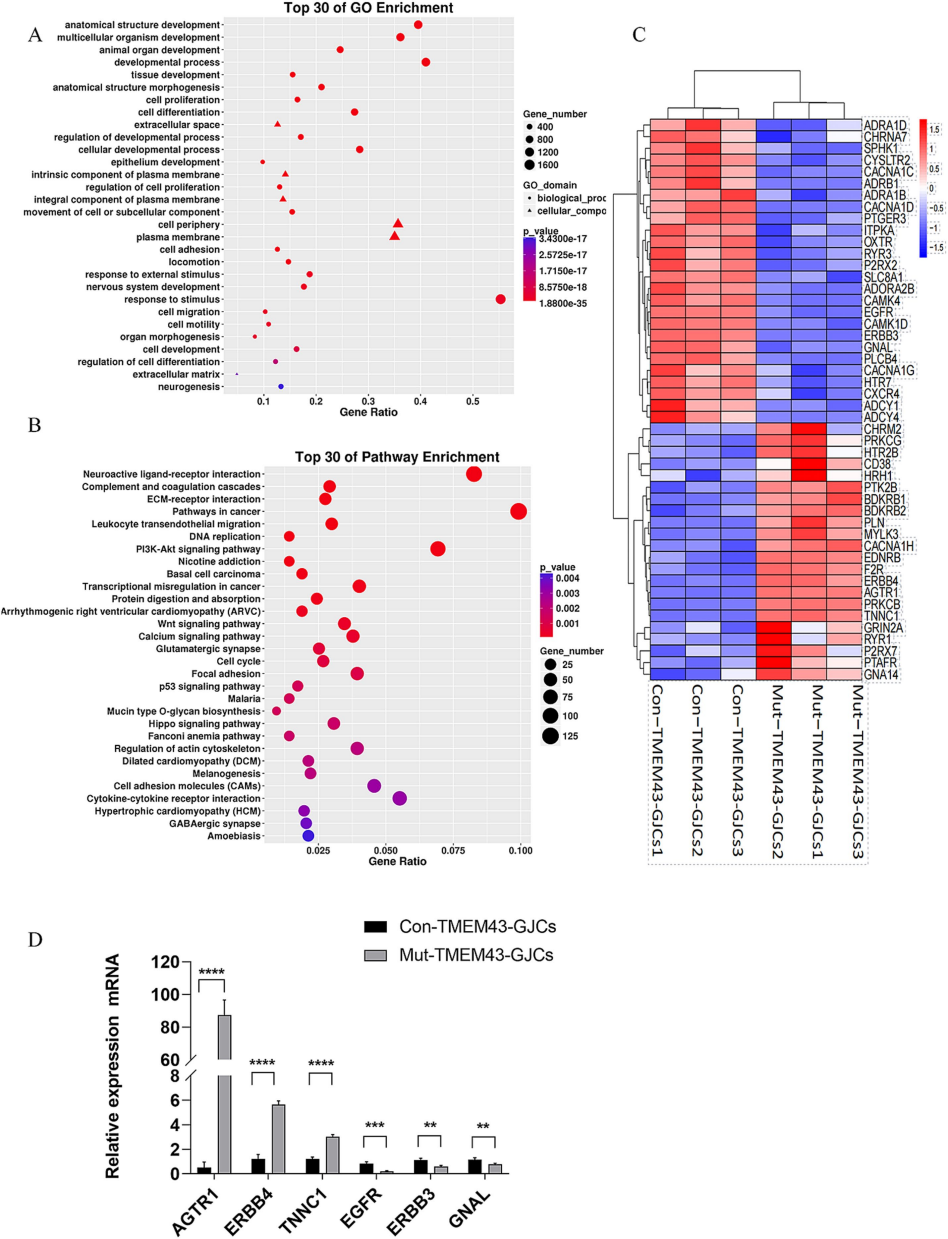
Differentially expressed gene analysis of Mut-TMEM43-GJCs and Con-TMEM43-GJCs. **(A)** The top 30 of GO enrichment analysis for DEGs. **(B)** The top 30 of KEGG enrichment analysis for DEGs. **(C)** Heat map analysis of differential genes in calcium signaling pathways in two groups of cells. **(D)** Quantitative RT-PCR analysis of the different expression genes, *n* = 3, (****p < 0.0001, ***p < 0.001, **p < 0.01).

To study the global gene expression (GGE) after differentiation, we performed RNA-seq analysis on Mut-TMEM43-GJCs and Con-TMEM43-GJCs cell lines to investigate difference of cellular differentiation casused by the TMEM43 mutation. On day 24 of differentiation, three replicate RNA samples from each group were subjected to RNA-seq. Heatmap of GGE showing differences in the expression patterns of most genes in the Mut-TMEM43-GJCs and Con-TMEM43-GJCs cell lines at the glia-like support cell stage (Figure 5A). Principal component analysis (PCA) of the transcriptome data showed similarity of duplicate samples within groups but significant differences between the two groups (Figure 5B). At the glia-like support cell stage, 1,212 genes showed differential expression between patients and controls (|log2FC|≧1, Q≦0.05). Among these, 647 genes were downregulated in expression and 565 genes were upregulated (Figure 5C). GO enrichment analysis showed that DEGs were enriched in association with cell proliferation, differentiation, extracellular space and adhesion, all necessary for inner ear development and maintenance of normal hearing (Figure 6A). Kyoto Encyclopedia of Genes and Genomes (KEGG) pathway analysis showed that DEGs are enriched in the PI3K-AKT signaling pathway and the calcium signaling pathway, both of which are required for hearing acquisition (Figure 6B; Ortolano et al., 2008). These DEGs are also enriched in the Wnt signaling pathway, cell adhesion and immunity, which are associated with cochlear development (Weng et al., 2023; Beaulac and Munnamalai, 2024). Calcium signaling pathways control mechanotransduction and synaptic transmission, and these are closely related to deafness (Galdieri et al., 2023). We further performed heat map analysis of differential genes in Calcium signaling pathways and found 48 differential genes, of which 22 were up-regulated and 26 were down-regulated (Figure 6C). We verified six genes by qRT-PCR. AGTR1, ERBB4, and TNNC1 expression was up-regulated, and EGFR, ERBB3, and GNAL expression was down-regulated consistent with the RNA-seq results (Figure 6D).

The current study identified a series of signaling pathways related to inner ear development and hearing loss through functional enrichment and KEGG analysis, including calcium signaling pathway, PI3K-AKT signaling pathway, Wnt signaling pathway, and cell adhesion, which may be involved in the pathogenesis of deafness caused by TMEM43 mutation.

## 4 Discussion

About 40% of ANSD is associated with genetic factors, such as autosomal recessive genes *OTOF* and *PJVK* (Varga et al., 2003; Delmaghani et al., 2006), autosomal dominant gene *OPA1* (Amati-Bonneau et al., 2009), and X-chromosome recessive gene *AIMF1* (Zong et al., 2015). The correlation of multiple genes with ANSD reflects the complexity of the molecular mechanism of ANSD. Synaptic sound coding in mammals occurs at the first auditory synapse between the inner hair cell and the afferent spiral ganglion neurons (SGN; Nayagam et al., 2011). This is a glutamatergic synapse. The opening of voltage-dependent calcium channels near the synapse at the base of the inner hair cell fuses the synaptic vesicle and releases glutamate presynaptically, transmitting sound signals to the SGN and ultimately to the auditory center. Presynaptic sites, the synapse itself, and postsynaptic and SGN lesions can all contribute to hearing loss. Our preliminary study identified a new deafness gene, TMEM43, whose mutation can lead to post-speech ANSD (Jang et al., 2021). TMEM43 is expressed predominantly in GLSs and is involved in the composition of gap junction channels, interacting with CX26 and CX30, which play important roles in the cochlea. The K^+^ channels are less expressed in GLSs, most resting membrane sites are mediated by gap junctions, and the GLSs gap junction network is involved in cochlear K^+^ recirculation (Zhao et al., 2006; Kikuchi et al., 2000). Gap junction intercellular communication is important for cell growth, development and homeostasis. Although animal experiments have investigated the function and role of TMEM43 in gap junction channels, however, the specific mechanism by which its mutation leads to human ANSD remains unknown. Patient-derived iPSCs are more conducive to modeling mechanisms of disease onset and progression *in vitro* (Inoue et al., 2014).

In this study, we successfully induced iPSCs from patients and their normal daughters and differentiated them into GLSs to study the effect of *TMEM43* mutations in GLSs and their functions. The karyotypes of these two iPSC cell lines were normal, and alkaline phosphatase staining, immunofluorescence staining of stemness genes, qRT-PCR of stemness genes, and teratoma experiments indicated pluripotency characteristics. Our results showed that Mut-TMEM43-iPSCs did not differ significantly in terms of pluripotency markers compared to Con-TMEM43-iPSCs. As previously studied, we subjected human iPSCs to floating and adherent cultures to generate TMEM43-GJCs characterized as cochlear support cells (Fukunaga et al., 2021; Fukunaga et al., 2019).

On the OV stage, our results showed that there was no significant difference in the morphology of the two groups of cells, and both groups of cells highly expressed the ear progenitor cell markers PAX2, PAX8, and GATA3 (Chen et al., 2012; Boddy et al., 2012). These results suggest that the Mut-TMEM43-iPSCs could differentiate to OVs as normal control in proliferation. Under the same culture conditions, the expression of *GJB2* and *GJB6* genes increased significantly on the 7th day in normal control (Beach et al., 2020; Defourny et al., 2019). However, the expression of *GJB2* and *GJB6* was not elevated in the *TMEM43* mutant group, suggesting that the *TMEM43* mutation may affect the generation and development to GLSs. Compared with iPSCs, the expression of *TMEM43* was not significantly changed in mutation and control groups, suggesting that such culture conditions did not increase the expression of TMEM43 during development, and it is also possible that the expression of TMEM43 is not obvious in the early stage of cochlear development. On GLSs stage, immunofluorescence and qRT-PCR further confirmed that TMEM43-GJCs highly expressed *GJB2*, *GJB6*, *TMEM43*, *SOX2*, *SPARCL1*, *KIAA1199*, *OTOR*, *MIA*, and these genes that are confirmed to be expressed in GLSs (Abe et al., 2003; Nie and Hashino, 2020). The immunofluorescence and qRT-PCR results showed that the expression of glia-like support cell-related marker genes was decreased at RNA and protein levels, suggesting that the *TMEM43* mutation may affect the differentiation of glia-like support cells. The significant difference in GLSs marker genes between the two groups of TMEM43-GJCs further suggests that the *TMEM43* mutation affects the production and development of GLSs, which might explain the delayed progressive hearing loss due to the *TMEM43* mutation (Jang et al., 2021). Our SL/DT experiments demonstrated that the *TMEM43* mutation resulted in decreased function of gap junctions between cells. Most studies suggest that dysfunctional gap junction intercellular communication between GLSs leads to hearing loss (Nickel and Forge, 2008; Zhu et al., 2015). Compared to controls, although our study did not reveal changes in the number and length of GJPs in mutant-TMEM43-GJCs, their function was significantly reduced, thus causing dysfunctional disorder in ANSD.

To better understand the biological and molecular processes of *TMEM43* in GLSs, we compared the differences in transcriptional profiles between Mut-TMEM43-GJCs and Con-TMEM43-GJCs. GO enrichment analysis showed that DEGs were associated with cell proliferation, differentiation, extracellular space and adhesion. Cell proliferation and differentiation are necessary to maintain inner ear development and normal hearing (Kopecky et al., 2013). Gap junction proteins form channels that connect cells directly and allow the exchange of small regulatory signals. Small molecule exchange has a major impact on intracellular metabolism (Kim et al., 2021). Communication conduits are formed between neighboring cells through gap junction proteins and extracellular space (Garcia-Vega et al., 2021). Cell adhesion molecules contribute to cell differentiation, regionalization of the otic capsule and extension of neural protrusions to ensure normal development of the inner ear and hair cells (Kelley 2003). Our transcriptome sequencing revealed changes in spectrin, and several studies have shown that spectrin has an essential role in cell adhesion and cell contact, suggesting that spectrin promotes morphogenesis through interactions with membrane proteins, actin, and adhesion molecules (Machnicka et al., 2019). Our results confirm that *TMEM43* mutations lead to changes in the extracellular space and cell adhesion. KEGG enrichment analysis similarly showed that DEGs were enriched in neural ligand-receptor interactions, PI3K-A signaling pathway and calcium signaling pathway. Impaired neuroactive ligand-receptor interactions have been reported to cause hearing loss (Maeda et al., 2021). The PI3K-Akt signaling pathway has been extensively studied in gap junction intercellular communication, such as the gap junction protein Pannexin 3 promotes osteoblast differentiation through activation of the PI3K-Akt signaling pathway (Ishikawa et al., 2011), platelet-derived growth factor AA promotes intercellular communication in chondrocytes by activating PI3K/Akt signaling-mediated expression of Cx43 (Xu et al., 2022). Several studies have also reported the relationship between the PI3K-Akt signalling pathway and hearing (Lai et al., 2023; Zhang et al., 2021). Calcium signaling pathways are more widely studied in the relationship between gap junction intercellular communication and hearing (Zhang et al., 2005). Connexin 26-associated hearing loss is thought to result from disruption of calcium signaling pathways. Our study suggests that TMEM43 mutations result in decreased gap junction function, which may lead to hearing loss through the PI3K-Akt signaling pathway and calcium signaling pathways.

Animal studies showed that the apical surface area of GLSs was not significantly altered in the early stage of TMEM43^KI^ mice, and young TMEM43^KI^ mice exhibit normal hearing (Jang et al., 2021). This is consistent with our patient-derived iPSC model, where we observed little effect of TMEM43 mutation on GLS development at early stages, mirroring the late onset of hearing loss in both the mouse model and patients. Previous studies have demonstrated decreased gap junction function in the TMEM43^KI^ mouse model (Jang et al., 2021), and our current study reaffirms this finding in patient-derived iPSC-differentiated GLSs. Additionally, we confirmed that there were no significant changes in GLS morphology or the number of GJPs. Moreover, we identified several hearing-related signaling pathways at the transcriptome level in patient-derived iPSC-differentiated GLSs, which paves the way for further mechanistic studies. In addition, the construction of patient-specific iPSC and the induction of GLSs provide the basis for subsequent drug screening. The limitation of our study is that we only established one iPSC line from an ANSD patient with a TMEM43 mutation. It would be helpful to generate additional patient-specific iPSC lines from different families. Moreover, potential variability in iPSC differentiation may have influenced our results. To address this, we could aim to differentiate iPSCs into more stable and reliable *in vitro* models, such as cochlear organoids to confirm our findings. In future studies, we may use pathway-specific inhibitors or activators to confirm the involvement of these pathways in TMEM43 mutant cells. Additionally, potential candidate genes identified through RNA-seq analysis in the ANSD model should be evaluated to identify targets that can protect GLSs, further advancing our understanding of potential therapeutic options.

## 5 Conclusion

This study provides a new perspective on the reduced function of *TMEM43* mutations in gap junction intercellular communication in GLSs by establishing an iPSC-based disease model based on an ANSD patient source. The TMEM43-p.(Arg372Ter) mutation affects the development and differentiation of GLSs, resulting in functional defects in gap junctions. Transcriptome sequencing and bioinformatics analyses show that *TMEM43* mutations may lead to hearing loss by affecting the PI3K-Akt signaling pathway and calcium signaling pathway resulting in decreased gap junction function, which may provide new ideas for drug screening and gene therapy. Consequently, the present study will contribute to a better understanding of the molecular mechanisms of ANSD caused by *TMEM43* mutations.

## Data Availability

The datasets presented in this study can be found in online repositories. The names of the repository/repositories and accession number(s) can be found below: https://www.ncbi.nlm.nih.gov/, with accession number PRJNA1191630.
