# Supplementary material for: Modeling of auditory neuropathy spectrum disorders associated with the TEME43 variant reveals impaired gap junction function of iPSC-derived glia-like support cells
Source: Front Mol Neurosci. 2025 Jan 6;17:1457874. doi: 10.3389/fnmol.2024.1457874 (PMC11743952; doi:10.3389/fnmol.2024.1457874)
Supplement: Supplementary file 1 [file Data_Sheet_1.ZIP › Supplementary/Table S1. The list of antibodies..docx]

Table S1. The list of antibodies.

|  | Antibody | Dilution | Company and Cat # |
| --- | --- | --- | --- |
| Primary antibodies  Secondary antibodies | Rabbit anti-NANOG  Rabbit anti-SOX2  Rabbit anti-OCT3/4  Mouse anti-SSEA-4  Mouse anti-TRA-1–60  Mouse anti-TMEM43  Rabbit anti-CX26  Rabbit anti-CX30  Goat anti-SPARCL1  Mouse anti-PAN  Mouse anti-CK18  Goat anti-Rabbit IgG (H + L) Cross-Adsorbed Secondary Antibody, Alexa Fluor™ 488  Goat anti-Rabbit IgG (H + L) Cross-Adsorbed Secondary Antibody, Alexa Fluor™ 568  Goat anti-Mouse IgG (H + L) Cross-Adsorbed Secondary Antibody, Alexa Fluor™ 488  Goat anti-Mouse IgG (H + L) Cross-Adsorbed Secondary Antibody, Alexa Fluor™ 568  Donkey anti-Goat IgG (H + L) Cross-Adsorbed Secondary Antibody, Alexa Fluor™ 488 | 1:200  1:400  1:400  1:400  1:200  1:50  1:50  1:100  1:100  1:100  1:100  1:500  1:500  1:500  1:500  1:500 | Abcam, ab21624  Abcam, ab97959  Abcam, ab19857  Abcam, ab16287  Abcam, ab16288  Santa, sc-365298  Thermo, 71-0500  Thermo, 71-2200  R&D, AF2728  Sigma, C2562-100UL  Thermo, MA5-12104  Thermo, A11008  Thermo, A11011  Thermo, A11001  Thermo, A11004  Thermo, A11055 |
|  |  |  |  |
