# Supplementary material for: Modeling of auditory neuropathy spectrum disorders associated with the TEME43 variant reveals impaired gap junction function of iPSC-derived glia-like support cells
Source: Front Mol Neurosci. 2025 Jan 6;17:1457874. doi: 10.3389/fnmol.2024.1457874 (PMC11743952; doi:10.3389/fnmol.2024.1457874)
Supplement: Supplementary file 1 [file Data_Sheet_1.ZIP › Supplementary/Supplementary.docx]

Figure S1. The episomal vectors were spontaneously lost in the Mut-TMEM43-iPSCs and Con-TMEM43-iPSCs at around 15–20 culture passages, as assessed by a PCR assay.

Figure S2. Immunofluorescence staining for GLSs markers CX30, CK18, PAN, bar = 20μm.

Table S1. The list of antibodies.

Table S2. The list of primer.
