# Supplementary material for: Modeling of auditory neuropathy spectrum disorders associated with the TEME43 variant reveals impaired gap junction function of iPSC-derived glia-like support cells
Source: Front Mol Neurosci. 2025 Jan 6;17:1457874. doi: 10.3389/fnmol.2024.1457874 (PMC11743952; doi:10.3389/fnmol.2024.1457874)
Supplement: Supplementary file 1 [file Data_Sheet_1.ZIP › Supplementary/Table S2. The list of primer..docx]

| Gene | Forward/Reverse primer (5′-3′) |
| --- | --- |
| SOX2 | TCACATGTCCCAGCACTACC/ CCCATTTCCCTCGTTTTTCT |
| OCT3/4 | CTGGGTTGATCCTCGGACCT/ CACAGAACTCATACGGCGGG |
| KLF4 | GATGAACTGACCAGGCACTA/GTGGGTCATATCCACTGTCT |
| NANOG | AAAGAATCTTCACCTATGCC/ GAAGGAAGAGGAGAGACAGT |
| GAPDH  PAX2 | GTGGACCTGACCTGCCGTCT/GGAGGAGTGGGTGTCGCTGT  GACTATGTTCGCCTGGGAGATTC/AAGGCTGCTGAACTTTGGTCCG |
| PAX8  GATA3  GJB2  GJB6  TMEM43  SPARCL1  KIAA1199  OTOR  MIA  AGTR1  ERBB4  TNNC1  EGFR  ERBB3 | TCAACCTCCCTATGGACAGCTG/GAGCCCATTGATGGAGTAGGTG  ACCACAACCACACTCTGGAGGA/TCGGTTTCTGGTCTGGATGCCT  ATCTGGCTCACCGTCCTCTTCA/TCGTAGCACACGTTCTTGCAGC  GAAACCACTCGCAAGTTCAGGC/AGGCTGCTTCAAAGATGATTCGG  TCTCCACCAAGTCTGGGGAT/TGTCCGAGCAACAAGGATGG  GTGAAGGCAACATGAGGGTGCA/GTTGGAGGACAAGTCACTGGATC  ACCGAGCACATTCCAACTACCG/GGCAGAGATGATTGAGAGGAACG  GCTGGTAAAAGAAAATGGAGCTGG/CACACGCTGTTCCTTGACCAAG  GCCAAGTGGTGTATGTCTTCTCC/CTGGTCCTCTCGGACAATGCTA  AAAGTCGGCACCAGATGAAGAAA/TTTGATCACCTGGGTCGAATTTG  ACATCCCACCTCCCATCTATACT/GGAGCTTCTGGAATTGTGCTAGT  CAGGAGATGATCGATGAGGTGGA/CAGACAGCTCCTCCTCAGATTTC  CTGGGTGCGGAAGAGAAAGAATA/CCAAAGGTCATCAACTCCCAAAC  TTGCCATCTTCGTCATGTTGAAC/GCCATTGTCCTTCACCACTATCT |
| GNAL | TCCTGCACGTCAATGGGTTTAAT/GGGCTATGCTCTTGATGTAGTCT |

Table S2. The list of primer.
