# Supplementary figures and images for: Modeling of auditory neuropathy spectrum disorders associated with the TEME43 variant reveals impaired gap junction function of iPSC-derived glia-like support cells
Source: Front Mol Neurosci. 2025 Jan 6;17:1457874. doi: 10.3389/fnmol.2024.1457874 (PMC11743952; doi:10.3389/fnmol.2024.1457874)

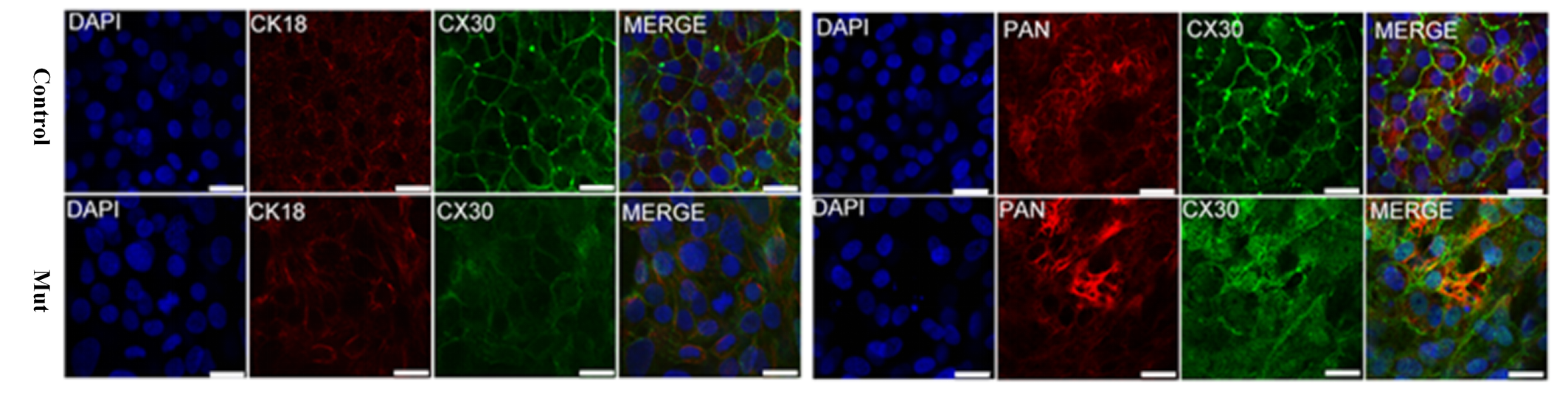

Supplement: Supplementary file 1 [file Data_Sheet_1.ZIP › Supplementary/Figure S2.tif]

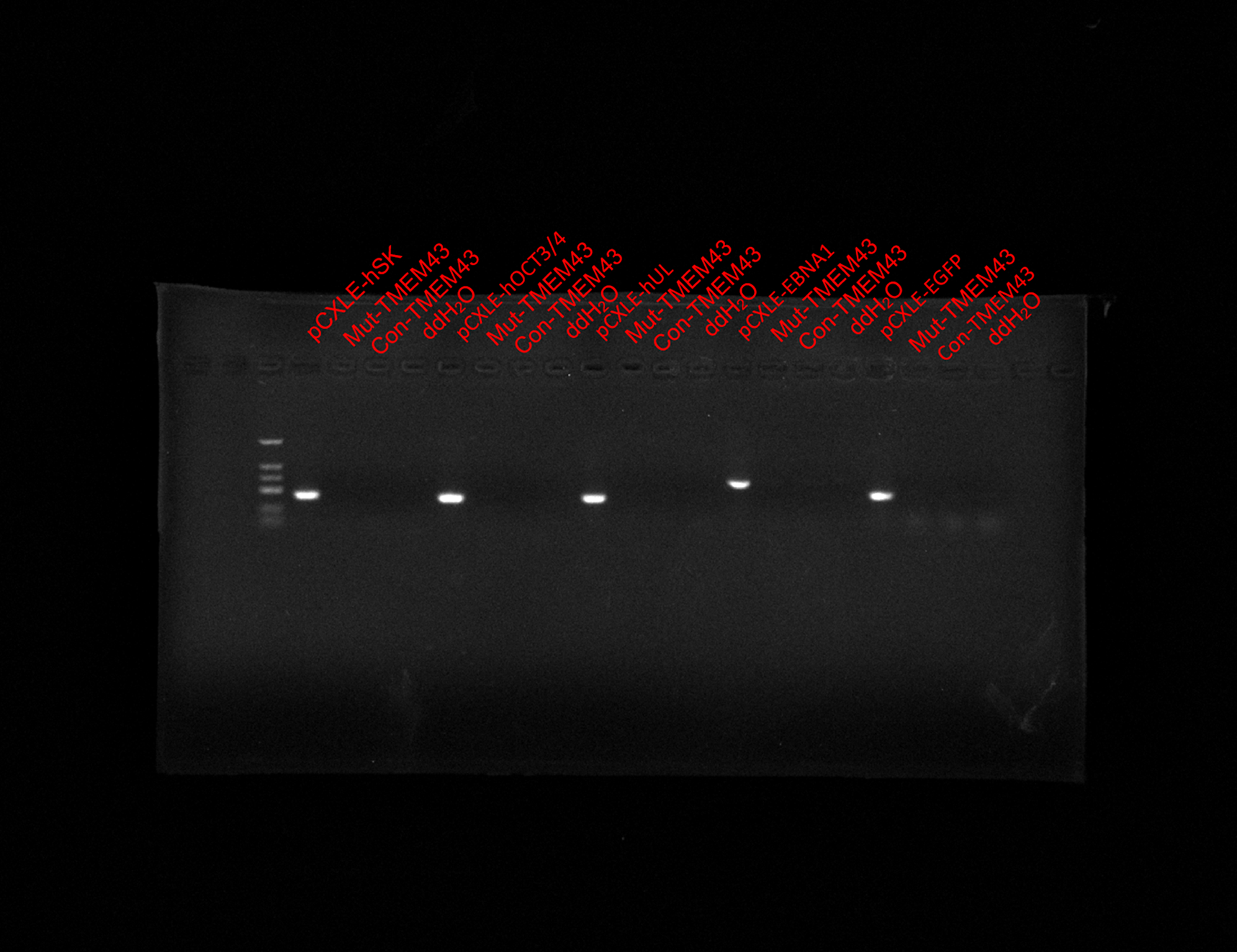

Supplement: Supplementary file 1 [file Data_Sheet_1.ZIP › Supplementary/Figure S1.tif]
